# Potential risk of plant viruses entering disease cycle in surface water in protected vegetable growing areas of Eastern China

**DOI:** 10.1371/journal.pone.0280303

**Published:** 2023-01-25

**Authors:** Xianping Zhang, Xiaohui Sun, Yongguang Liu, Ning Qiao, Xueyu Wang, Dan Zhao, Kaijie Shang, Xiaoping Zhu

**Affiliations:** 1 College of Plant Protection, Shandong Agricultural University, Taian, Shandong, China; 2 Facility Horticulture Laboratory of Universities in Shandong, Weifang University of Science and Technology, Shouguang, Shandong, China; Universidade Lisboa, Instituto superior Técnico, PORTUGAL

## Abstract

With the expansion of protected vegetable growing areas (PVGAs), viral plant diseases have become more prevalent, causing severe economic losses to the vegetable production industry in China. At present, researches on plant viruses mainly focus on plants, but there is only a few reports on the species of viruses in surface water from PVGAs. The surface water samples in PVGAs are representative to a certain extent, which has an important reference value for studying the characteristics of plant viruses in surface water. The purpose of this study was to identify the diversity and the possibility of entering disease infection cycle of plant viruses in water samples collected from PVGAs in eastern China. A total of 144 water samples were collected, and eight plant viruses including tobacco mosaic virus (TMV, 8.33%), cucumber green mottle mosaic virus (CGMMV, 33.33%), pepper mild mottle virus (PMMoV, 6.94%), cucumber mosaic virus (CMV, 0.69%), tomato masaic virus (ToMV, 3.47%), tomato mottle mosaic virus (ToMMV, 0.69%), tomato chlorosis virus (ToCV, 4.17%), and tomato yellow leaf curl virus (TYLCV, 5.56%) were examined using RT-PCR and PCR. The species of viruses in surface water varied greatly by location. CGMMV, TMV, ToCV, ToMV, ToMMV, and TYLCV were identified in Shandong, a northern part of Eastern China, whereas only PMMoV was found in Shanghai, a southern part of Eastern China. After healthy tobacco plants were inoculated with the concentrated solutions of TMV, ToMV, CGMMV, and PMMoV, could cause disease in healthy tobacco, indicating that the plant viruses in the concentrated solution have the infectivity, and the plant viruses in surface water have the possibility of entering the infection cycle of disease. The results will improve the understanding of the potential risks of waterborne disease transmission.

## Introduction

There are many pathogenic microorganisms in surface water, such as plankton, bacteria, and viruses [1–6]. Viruses have been widely considered the most dangerous potential pathogens in surface water [7]. As a carrier of plant viruses, water plays a critical role in the transmission of plant diseases. Studies on the existence and pathogenesis of animal viruses in aquatic environments are relatively mature, but the harmful effects of plant viruses in water have not attracted widespread attention [8]. Plant viruses in surface water have a wide range of sources. According to the source organisms, they are mainly divided into two categories: one is plants, whose roots can release plant viruses into surface water; the other is animals, mainly vertebrates (ie, humans and other plant-eating animals) because their digestive tracts contain many undigested plant viruses that can be released into and contaminate surface water [9,10]. Therefore, there may be a large number of plant viruses in surface water, which may cause plant disease by infecting plants. It is necessary to conduct in-depth research on plant viruses in water bodies around plants.

As a country with a large agricultural production and population, PVGAs are the main places for the production of vegetables in China. Shandong has a long history of vegetable planting and plays an important role in the national vegetable supply. Shanghai is one of the largest cities in Eastern China with a dense population and its demand for fruits and vegetables is high, so its PVGAs also play a crucial role. Surface water is the main source of agricultural irrigation water, due to the scarcity of water resources, especially in developing countries [11]. This appears to be economically profitable, but the quality of surface water used for irrigation varies and can have significant negative impacts on soils, crops, even threat to the health of farmers and consumers [12,13]. Although surface water containing high levels of organic matter and nutrients can increase crop yields, the presence of plant viruses may increase the risk of disease transmission, as many studies have found that suspensions containing plant viruses such as TMV, potato virus X (PVX), tomato bushy stunt virus (ToBSV) and CMV, can infect the roots of healthy plants [14,15]. Nevertheless, there is little information on the microbiological risks of using surface water for irrigation, especially in most developing countries [16], which may eventually lead to outbreaks of plant viral diseases in certain areas and could adversely affect the development of the vegetable industry [17]. The surface water samples around PVGAs in China are representative to a certain extent. Vegetables widely cultivated in Shandong and Shanghai include those that belong to the *Solanaceae* and *Cucurbitaceae* families among other common vegetable varieties. The *Tobamovirus*, *Begomovirus*, *Potyvirus*, *Potexvirus*, *Polerovirus*, *Tospovirus*, *Closteroviride*, *Cumovirus* and *Crinivirus* have a high detection rate in PVGAs [18].

Waterborne plant viruses can destroy entire crops, leading not only to high economic losses but also to food shortages [19]. Waterborne human pathogenic viruses can remain on infected plants and cause infections when consumed [20], while plant viruses can cause huge crop losses [21]. This is particularly problematic in hydroponics systems, where they can multiply and cause further crop infection [22]. Water has been shown to mediate the spread of various plant viruses, including resilient tobamoviruses such as CGMMV, TMV, and two other important plant viruses: pepino mosaic virus (Potexvirus, Alphaflexiviridae), potato virus Y (PVY) (Potyvirus, Potyviridae) [23,24]. This further shows that the harm of plant viruses in the surrounding water to plants needs to be widely paid attention to it. However, there is no research on the distribution of plant viruses in the water around PVGAs and whether the existing plant viruses can affect vegetables in the PVGAs.

Recently, research on plant viruses in water has gradually developed, owing to the successful study of animal viruses in water [25]. The advent of high-throughput sequencing has revolutionized the field of environmental virology, enabling a better understanding of viral diversity and distribution in nature [26]. However, there are relatively few studies on surface water plant viruses in China, and there is no research on the species and distribution of surface water plant viruses in PVGAs. In this study, common plant viruses were detected in water samples from PVGAs in Shandong and Shanghai using genus-specific primers. An inoculation test was used to examine the potential pathogenicity of plant viruses and to demonstrate the possibility of plant viruses entering the plant disease cycle. The results of this study can aid in predicting the agricultural burden of water-borne diseases and the pathogenicity of plant viruses and can be used to guide farmers in irrigating water properly, thus reducing the risk of plant virus transmission in surface water.

## Materials and methods

### Sample collection

Shandong and Shanghai, two of the most important cities in Eastern China, have huge vegetable planting areas. Field investigation shows that the types and quantities of surface water in different PVGAs vary greatly. We took surface water samples within 2 kilometers of the PVGAs as the research object, and collected water samples from the nearest distance to the PVGAs for further research. From July 2015 to January 2018, 144 water samples were collected from Shandong and Shanghai, including 82 samples of river water, 59 samples of agriculture drainage ditch warter, and 3 samples from springs (Table 1). The sampling areas were characterized by a high population density and extensive agricultural land. At each sampling location, source water samples (>10 L each) were collected and transported to the laboratory within two days. The samples were stored at <10°C during transportation and processed for virus concentration immediately after delivery.

**Table 1:** Sample collection information.

Sampling site	River	Agriculture drainage ditch	Spring	Total
**Jinan, Shandong**	**12**	**2**	**3**	**17**
**Weifang, Shandong**	**15**	**4**	**0**	**19**
**Yantai, Shandong**	**4**	**2**	**0**	**6**
**Linyi, Shandong**	**5**	**7**	**0**	**12**
**Liaocheng, Shandong**	**9**	**8**	**0**	**17**
**Dezhou, Shandong**	**5**	**2**	**0**	**7**
**Heze, Shandong**	**4**	**2**	**0**	**6**
**Binzhou, Shandong**	**3**	**1**	**0**	**4**
**Weihai, Shandong**	**4**	**0**	**0**	**4**
**Taian, Shandong**	**3**	**12**	**0**	**15**
**Zibo, Shandong**	**8**	**4**	**0**	**12**
**Jining, Shandong**	**3**	**2**	**0**	**5**
**Shanghai**	**7**	**13**	**0**	**20**
**Total**	**82**	**59**	**3**	**144**

### Concentration of viruses

Viruses were extracted from the water sources using secondary concentration methods described previously Kuroda [21]. As described below. Firstly, a 2 L water sample was filtered into a beaker with double gauze, after which 8 mL Triton X-100 was added. Then, 120 g PEG 6000 and 20 g NaCl were added and stirred thoroughly for more than 4 h. After being left to rest overnight, the liquid was centrifuged at 8000 rpm for 20 min and resuspended with 100 mL 0.2 mol L^-1^ phosphate buffer saline (PBS) at pH 7.2 and 0.5 mL Triton X-100. It was then stirred slowly for approximately 3 h at 4°C, after which 0.5 mL of Triton X-100 was added to it. Then it was stirred slowly for an additional 3 h at 4°C to ensure that it was mixed well. Finally the supernatant collected after centrifugation at 5000 rpm at 4°C for 25 min was discarded after centrifugation at 100000 rpm for 1.5 h, and 200 μL of 0.01 mol L^-1^ PBS (pH8) was added to resuspend the remaining sediment, namely the extracted virus particles.

### Viral genome extraction and reverse transcription

An EasyPure®Viral DNA/RNA Kit (TransGen Biotech, Beijing, China) was used to extract viral DNA and RNA. A high-capacity cDNA reverse transcription kit (TransGen Biotech, Beijing, China) and a PCR Thermal Cycler Dice (TP600) (TaKaRa, Japan).were used to perform the reverse transcription of RNA samples to single-stranded cDNA. The resulting cDNA samples were then subjected to a conventional PCR and RT-PCR.

### Amplification of plant viruses

A 25 μL mixture containing 1 μmol L^-1^ primers, 0.025 units μL^-1^ Taq DNA Polymerase (Vazyme, Nanjing China), 1 mM MgCl_2_, 0.1 mM dNTPs (dATP, dCTP, dGTP, dTTP), 2 μL DNA template, and add ddH_2_O to 25 μL. The PCR program was 94°C for 3 min, followed by 32 cycles of 94°C for 30 s, 55°C for 30 s, and 72°C for 1 min, and 72°C for 5 min. The PCR amplification products were analyzed by electrophoresis on a 1% agarose gel, which was stained with EB and observed under a UV transilluminator (Bio-Rad, Hercules, CA, USA). All primer sequences used in this study are shown in Table 2.

**Table 2:** Primers used for detection of the main vegetable viruses in this study.

Primer name	Sequence (5’-3’)	size/bp
**Tobamovir-F**	**GTCGCSGAWTCKGATTCGTWTTA**	**709**
**Tobamovir-R**	**TGGGCCSCWACCGGSGGTWMC**	
**Potyvirus-F**	**GGNAAYAAYAGYGGNCARCC**	**1700**
**Potyvirus-R**	**GTTTTCCCAGTCACGAC**	
**Potexvirus-F**	**GTTTTCCCAGTCACGAC**	**2500**
**Potexvirus-R**	**GGNGARGGNCCNACNTT**	
**Tospovirus-F**	**ATGGGDATNTTTGATTTCATG**	**1180**
**Tospovirus-R**	**TCATGCTCATSAGRTAAATYTCTCT**	
**Closteroviride-F**	**GGNTTAGANTTCGGNACNAC**	**517**
**Closteroviride-R**	**TCAAANGTNCCNCCNCCNAA**	
**Crinivirus-F**	**GCYCCSAGRGTKAATGA**	**550**
**Crinivirus-R**	**ACCTTGRGAYTTRTCAAA**	
**Cucumovirus-F**	**YASYTTTDRGGTTCAATTCC**	**950**
**Cucumovirus-R**	**GACTGACCATTTTAGCCG**	
**Begomovirus-F**	**TGYGARGGICCITGYAARGTYCARTC**	**1200**
**Begomovirus-R**	**ATHCCMDCHATCKTBCTlTGCAATCC**	
**Poleroviruses-F**	**CGTCTACCTATTTSGGRTTN**	**1420**
**Poleroviruses-R**	**TGYTCYGGTTTTGACTGG**	
**CGMMV-F**	**ATGGCTTACAATCCGATC**	**486**
**CGMMV-R**	**CTAAGCTTTCGAGGTGGTAGCCTC**	
**TMV-CP-F**	**TCGAATTCACCATGTCTTACAGTATCAC**	**499**
**TMV-CP-R**	**TGGGATCCTCAAGTTGCAGGACCAGAGG**	
**ToMV-F**	**CAAATCCTCAAAAAGAGGTCCG**	**670**
**ToMV-R**	**CAAACTTTATATTTCAGCACCTATGCA**	
**PMMoV-F**	**ATGGCATACACAGTTACCAGT**	**474**
**PMMoV-R**	**TTAAGGAGTTGTAGCCCACGTA**	
**ToCV-F**	**GGCTAATCCTAATCGATCTTCCAGT**	**570**
**ToCV-R**	**GCGTTTCTTTTCATAAGCAGGTTC**	
**TY2-F**	**CTCCAAAATCAATGAAGTCTCC**	**751**
**TY2-R**	**GGGCTCGTAAGTTTCCTC**	
**ToMMV-F**	**AGAGAGATGGCGATAGGTTAAC**	**1018**
**ToMMV-R**	**CTGCAGTCATAGGATCTACTTC**	
**CMV-F**	**GTCACGGACTATGATAAG**	**260**
**CMV-R**	**TTACGCATGTCGCCGATAT**	

### Inoculation test of virus concentrate

Seedlings of the tested plants were grown in a cultivation chamber at 24 ± 2°C under light (16 h) and 20 ± 2°C in the dark (8 h), with a relative humidity of 75% ± 5%. The mechanical inoculation method was applied to the purified *Tobamovirus* solution, as a paragon to verify the infectivity of plant viruses in surface water, as *Tobamovirus* is the predominant type of virus present in it. The virus-purified solution was mixed with an equal volume of 0.1 M PBS (pH 7.2), and the tobacco leaves were gently rubbed, while controls with 0.1 M PBS. Six replicates were used for each treatment. No other viruses were transmitted during the experiment. Infectivity of the test plants was confirmed on newly developed leaves by RT-PCR detection roughly 10 days after inoculation.

## Results

### Main plant virus species and detection results in surface water samples

**Fig 1:**
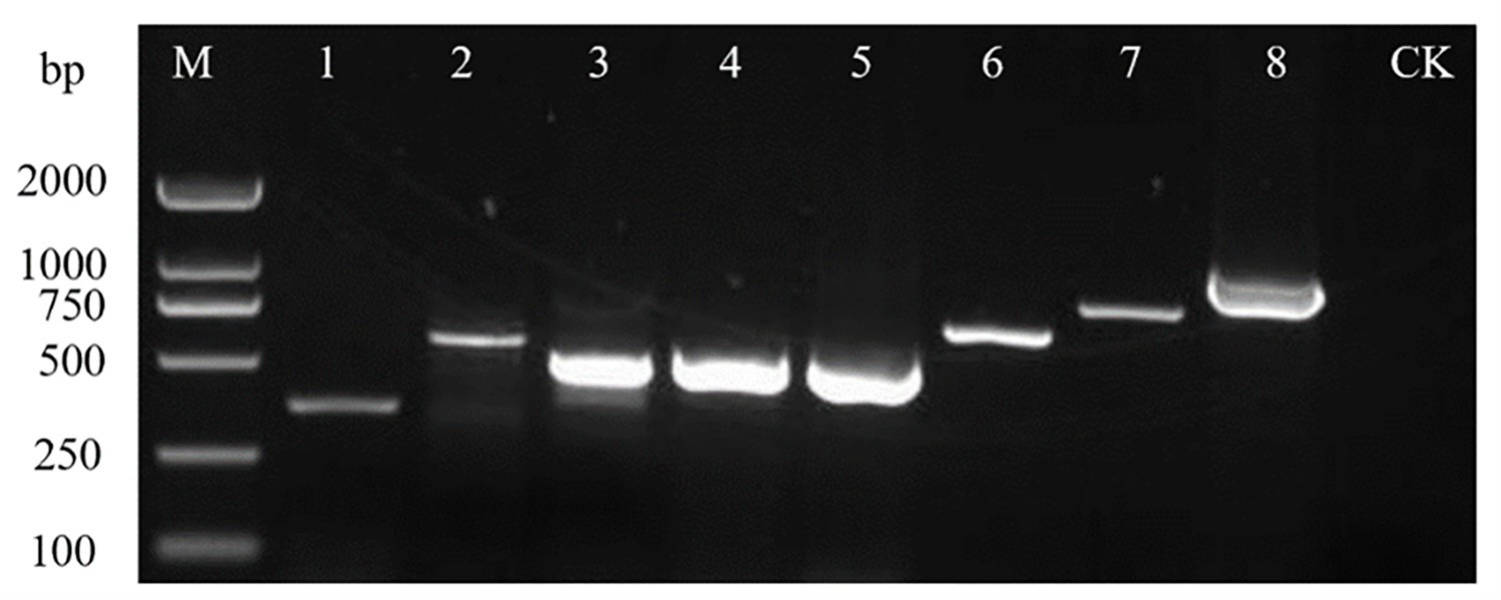
Electrophoresis results of virus detected in surface water. M: Trans2K® DNA Marker; 1–8: CMV; PMMoV; CGMMV; TMV; ToCV; ToMV; TYLCV; ToMMV; CK: Negative control.

**Fig 2:**
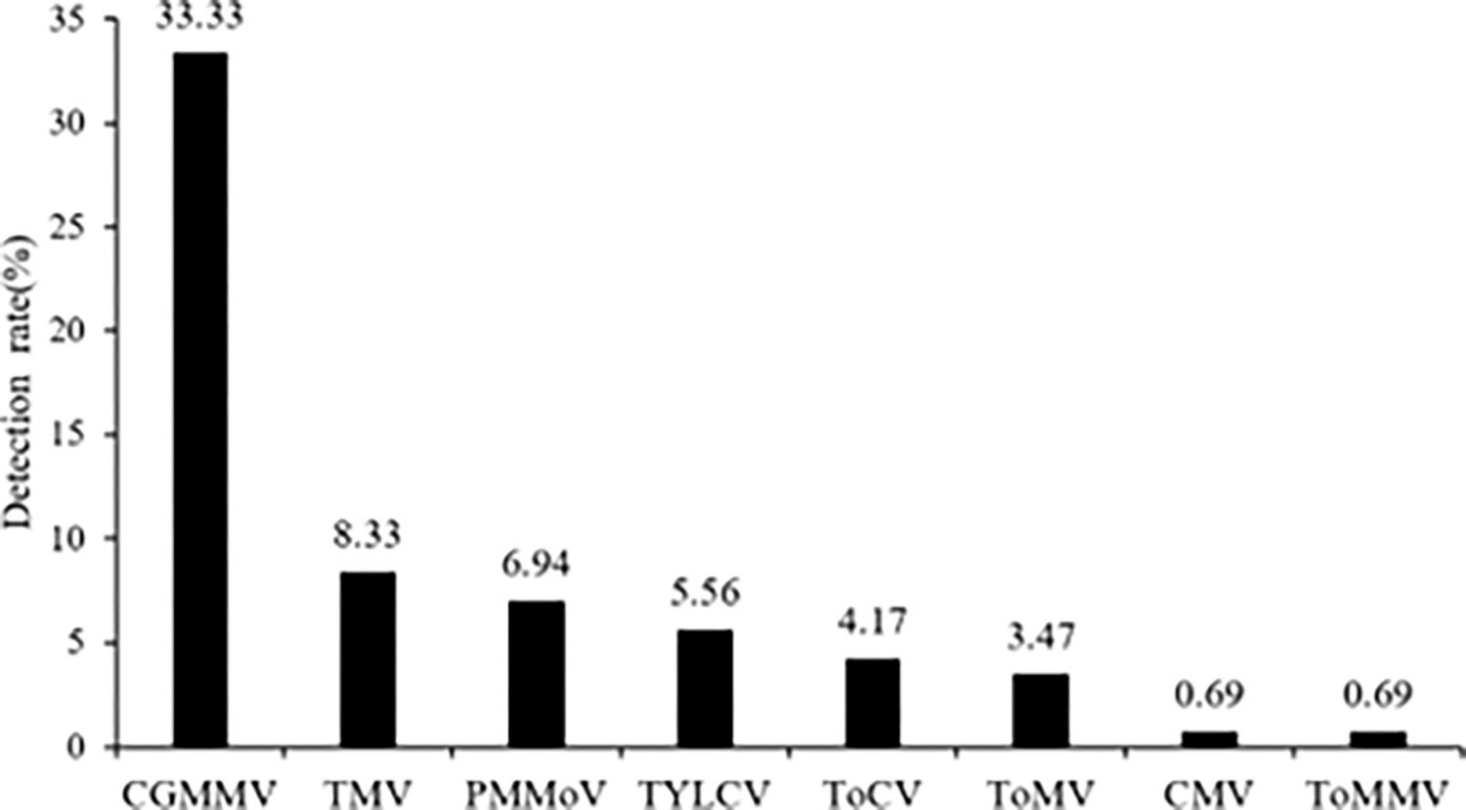
Detection of each virus in surface water.

As shown in Figs 1 and 2, the results of the PCR tests that were conducted on the collected water samples showed a total of eight plant viruses were detected, including seven types of RNA viruses: CMV, PMMoV, CGMMV, TMV, ToCV, ToMV, and ToMMV, while a DNA viruses, TYLCV, was detected. Plant viruses were detected in 68 of the samples, yielding a detection rate of plant viruses of 47.22%. The detection rate of CGMMV was the highest (33.33%), followed by that of TMV (8.33%). The detection rates of CMV and ToMMV were the lowest (0.69% each).

### Virus species detected in different regions

As shown in Table 3, there were large differences in virus types across different regions: CGMMV and other viruses were detected in the water samples from Shandong, while only PMMoV was detected in the water samples from Shanghai, which suggests that the distribution of the plant viruses in surface water varies greatly.

**Table 3:** Species of plant viruses in surface water from different regions.

Location	Virus species
**Jinan, Shandong, China**	**CGMMV+TMV+ToCV+ToMV+ToMMV+TYLCV**
**Weifang, Shandong, China**	**CGMMV+ TMV+CMV+PMMoV**
**Yantai, Shandong, China**	**PMMoV+ToCV+TYLCV**
**Linyi, Shandong, China**	**CGMMV+ PMMoV**
**Liaocheng, Shandong, China**	**CGMMV+TYLCV**
**Dezhou, Shandong, China**	**CGMMV+TYLCV**
**Heze, Shandong, China**	**TMV+CGMMV**
**Binzhou, Shandong, China**	**CGMMV+ToCV**
**Weihai, Shandong, China**	**CGMMV**
**Taian, Shandong, China**	**CGMMV**
**Zibo, Shandong, China**	**ToCV**
**Jining, Shandong, China**	**-**
**Shanghai, China**	**PMMoV**

“-” means no plant viruses have been detect.

The results showed that in 82 river water samples, there were 43 CGMMV positive samples (52.44% detection rate), while among 59 samples of agriculture drainage ditch, 7 CGMMV positive samples (11.86% detection rate). These results suggest that CGMMV is ubiquitous in surface water and could be a potential candidate for evaluating surface water pollution indicators in the future.

Through statistical comparison (Table 3) of the water samples from Shandong, we found that the largest number of virus species was detected in the water samples from Jinan, with a total of six viruses, including CGMMV, TMV, ToCV, ToMV, ToMMV, and TYLCV. A significant difference was observed between the water samples of Shandong and Shanghai, with only PMMoV being detected in the water samples from Shanghai.

In addition, from Table 3 shows that the virus species in the water samples collected from the Shandong area are rich and the distribution is quite different. The most virus species were detected in the water samples in Jinan, and six viruses were CGMMV, TMV, ToCV, ToMV, ToMMV, and TYLCV. Four viruses (CGMMV, TMV, CMV, and PMMoV) were detected in the water samples from Weifang. While three viruses (ToCV, PMMoV, and TYLCV) were detected in those from Yantai. Two viruses (CGMMV and ToCV) were detected in the water samples from Binzhou. Two viruses (CGMMV and TYLCV) were detected in the water samples from Liaocheng and Dezhou. Two viruses (CGMMV and TMV) were detected in the water samples from Heze, as well as in the samples from Linyi, (CGMMV and PMMoV). Only CGMMV was detected in the water samples from Taian and Weihai, while only ToCV was detected in the water samples from Zibo. It is noteworthy that two plant viruses, including TYLCV and ToCV, were detected simultaneously in the Baishi Spring in Jinan, indicating that plant viruses existed abundantly, and their distribution varied greatly, in the water samples from Shandong.

**Fig 3:**
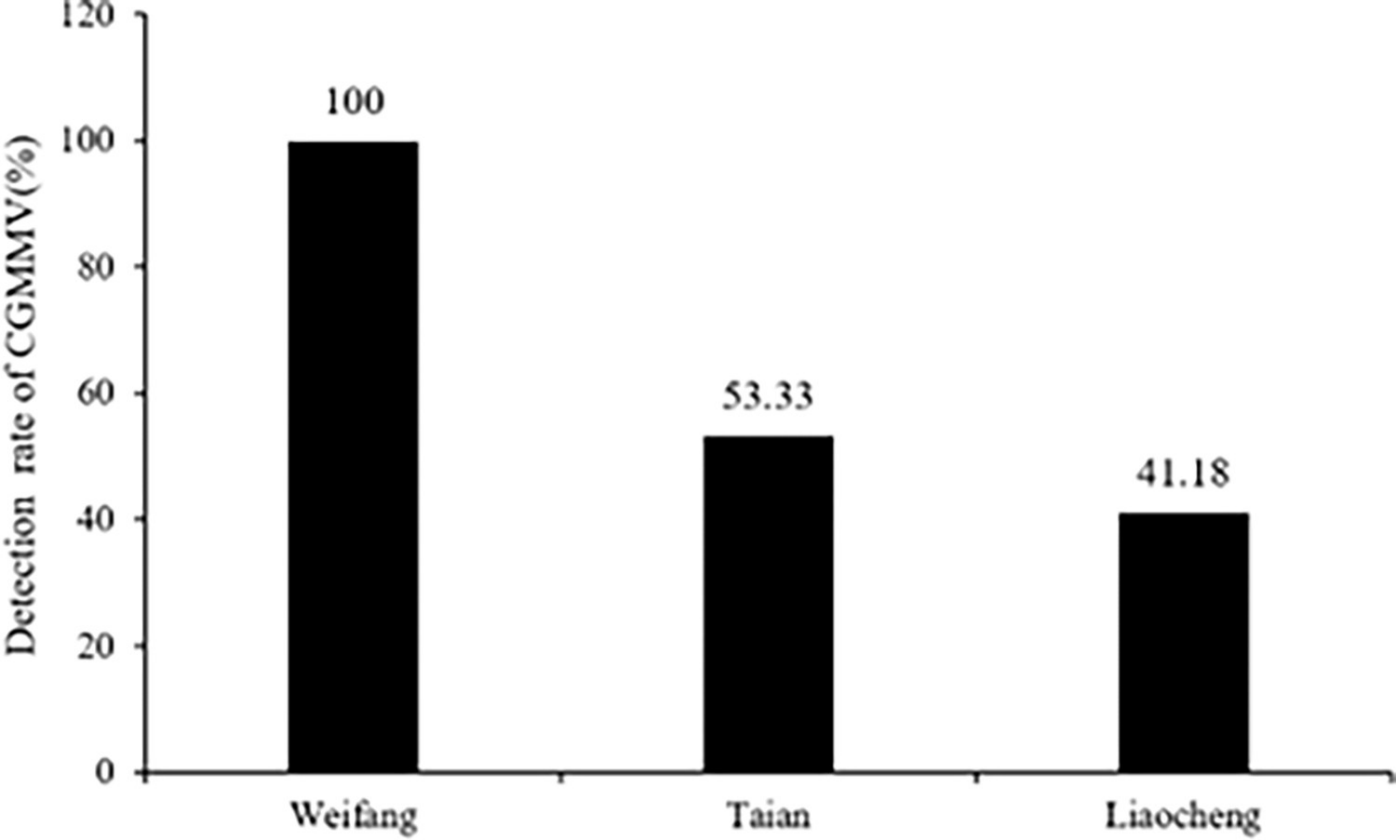
Detection of CGMMV in different PVGAs in Shandong.

According to the results from the analyses of the water samples in and around the main PVGAs in Shandong (Weifang, Taian, and Liaocheng), the detection rate of CGMMV was high in the surface water around them. The detection rate of CGMMV was 100% (19/19) in all water samples from Weifang, 53.33% (8/15) in the water samples from Taian, and 41.18% (7/17) in the water samples from Liaocheng (Fig 3).

### Inoculation test of concentrated virus

**Fig 4:**
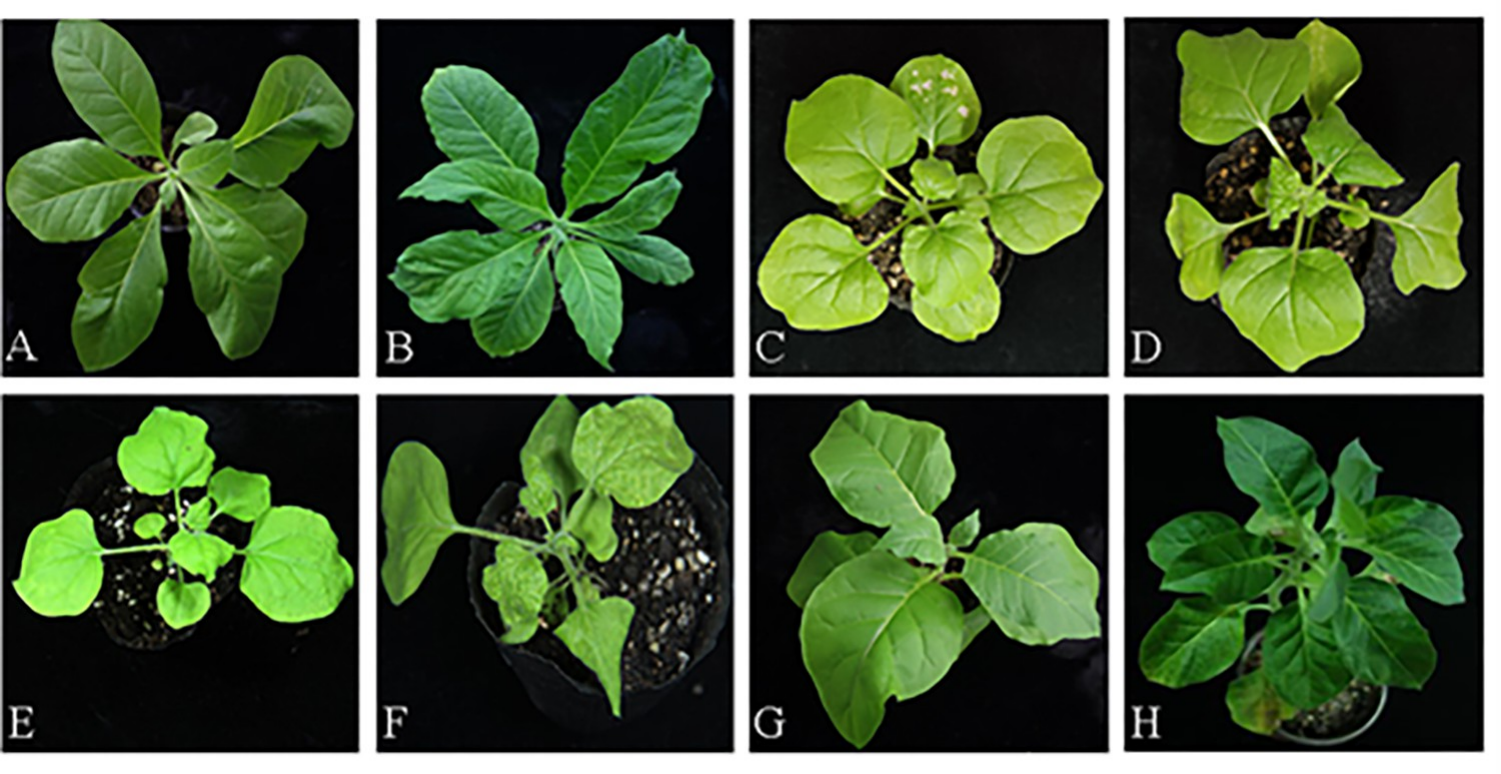
Inoculation test. A、B: Inoculation test of TMV. A: Control group; B: Experimental Group; C、D: Inoculation test of ToMV. C: Control group; D: Experimental Group; E、F: Inoculation test of CGMMV. E: Control group; F: Experimental Group; G、H: Inoculation test of PMMoV. G: Control group; H: Experimental Group.

The concentrated solution of *Tobamovirus* (TMV, ToMV, CGMMV, and PMMoV) and 0.1 M PBS were mixed in a 1:1 ratio and then inoculated into tobacco seedlings at 4–6 leaves stage. After 5–7 days, the deformities of the leaves and mosaicism were obvious (Fig 4).

### Molecular detection after inoculation with concentrated virus

**Fig 5:**
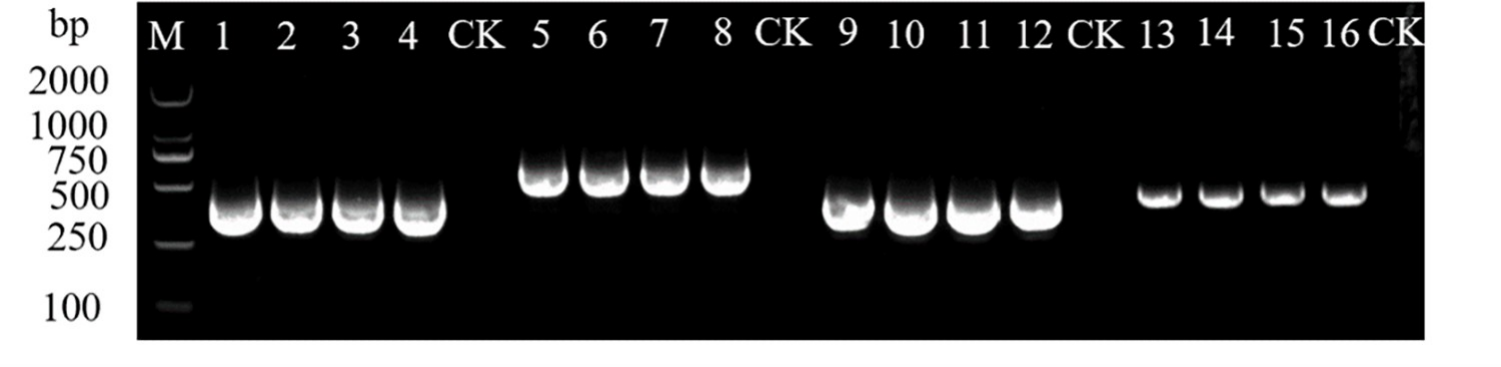
RT-PCR test results of inoculated tobacco. M: Trans2K® DNA marker; CK: Negative control; 1–4: Tobacco samples inoculated with TMV concentrate; 5–8: Tobacco samples inoculated with ToMV concentrate; 9–12: Tobacco samples inoculated with CGMMV concentrate; 13–16: Tobacco samples inoculated with PMMoV concentrate.

RT-PCR was performed on the inoculated tobacco after 5–7 days to verify whether plant viruses in surface water can enter the plant disease cycle (Fig 5).

**Fig 6:**
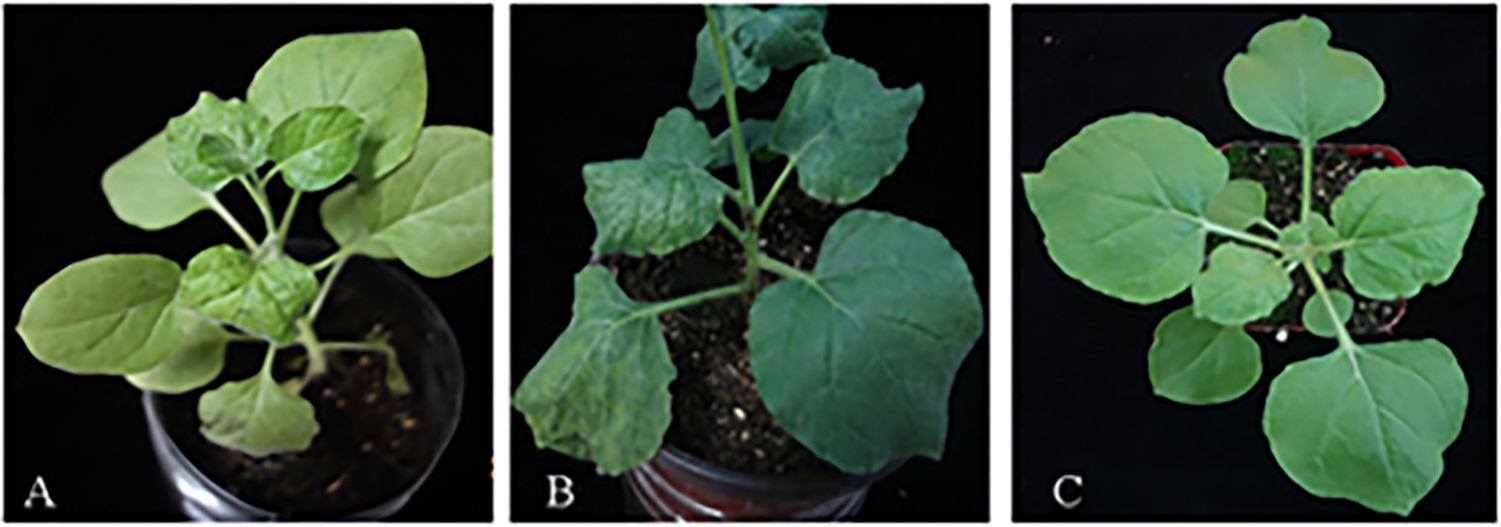
Irrigation test of tobacco. A: Symptoms of tobacco watering with TMV-carrying water after 14 days. B: Symptoms of tobacco watering with TMV-carrying water after 28 days. C: Symptoms of tobacco watering with sterile water.

**Fig 7:**
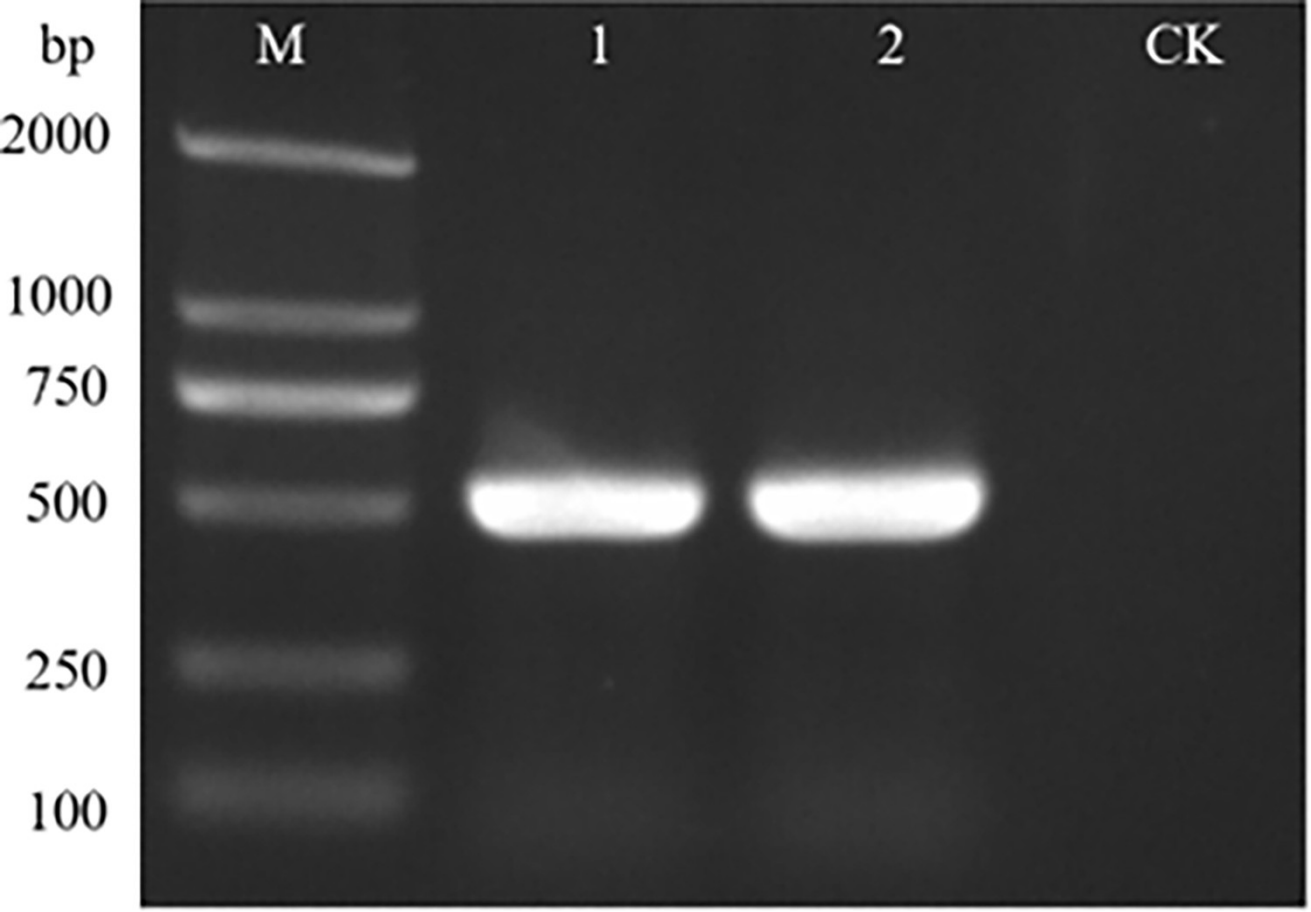
RT-PCR test results of irrigated tobacco. M: Trans2K® DNA marker; 1–2: Tobacco samples watered with water samples containing TMV; CK: Negative control.

A typical plant virus, TMV, was selected as a model for the root irrigation test, and the plants showed typical symptoms of TMV after two weeks (Fig 6A), and PCR result confirmed the presence of TMV (Fig 7).

## Discussion

Shandong and Shanghai are representative of Eastern China (Shandong being a northern region, and Shanghai being a southern region of Eastern China). The distribution of plant viruses in surface waters is quite different in these two regions, which may be related to the differences in water quality between them. Shanghai is industrially developed, and the water samples collected from there were dark in color. There were relatively more algae in the water from Shanghai, and the detection rate of plant viruses in those water samples was relatively low. The species of plant viruses present in the water may be related to the algae present in it.

The plant virus, PMMoV has been shown to be associated at high concentrations with human feces, making it an abundant virus in surface water [27,28], probable, in most countries and regions where peppers and pepper-based products are consumed. Previous studies have reported the presence of PMMoV in the feces of birds such as geese, seagulls, and chickens. Among these, the feces of black swans (90%) and seagulls (50%) show the highest incidence of PMMoV [2,25,29]. This may be related to their diets of aquatic plants and animals. In recent years, researchers have discovered that PMMoV is distributed globally, with multiple sources of water, more than human pathogenic viruses, and with no obvious seasonal fluctuations. It has, therefore, been proposed to be used as an indicator of water pollution [30–33]. CGMMV is widely detected in aquatic environments of Eastern China, especially in the aquatic environment around the PVGAs of Shandong, while PMMoV is detected in large numbers in Shanghai. This difference may be due to variations in geography, climate, and diet. Shandong is the most prominent area producing vegetables that belong to the *Solanaceae* and *Cucurbitaceae* families, where vegetables are produced all year round, and the incidence of plant virus diseases is relatively serious. This is also an important reason for the high detection rate of CGMMV in Shandong. Continuous monitoring of the waters near the PVGAs in China revealed that CGMMV has a certain degree of stability in aquatic environments. Therefore, it is necessary to share these findings with farmers to enable them to use water resources for irrigation safely and prevent an epidemic of viral diseases in PVGAs that have a high detection rate of CGMMV.

CMV is relatively unstable in aquatic environments, and its infectivity is lost within days or hours at room temperature. This may explain the low detection rate of CMV in surface water [34]. ToCV and TYLCV are phloem-limited and are spread by vector insects, which cause serious damage to vegetables in the Shandong area. However, their detection rate in surface water is not high, which may be due to their low virus titer in the host plants.

Water as a virus transmission vector is closely relevant in agriculture production, as 70% of all water is used for irrigation [35]. The risk of transmission in agricultural irrigation water is alarming, and the inactivation method of viruses in water has been a hot and difficult topic. Common methods for inactivating viruses in water bodies include chlorine disinfection, ozone disinfection and ultraviolet disinfection. Recent studies have found that hydrodynamic cavitation (HC) can successfully inactivate PVY in water samples [36]. For various reasons, these methods are only used in closed water systems, especially in the increasing use of closed irrigation systems. Due to the complexity of the field environment and the limitation of equipment scale, it is difficult to apply a single method to the disinfection treatment of field surface water. We believe that in the near future, with the synergistic use of multiple treatment methods and the continuous expansion of equipment scale, significant progress can be made in the disinfection of field surface water, which will escort the safety of food production.

In fact, there are many other plant viruses in surface water. Owing to the interplay of various factors, only a small number of the plant viruses in surface water have been detected. Plant viruses present in water pose a potential threat to agricultural production; therefore, it is necessary to understand water contamination by plant viruses and the potential dangers of their spreading. Research on purification technologies that can be used to prevent the contamination of water sources by viruses and to remove them is the primary means of preventing large-scale epidemics of plant viral diseases transmitted by water, which will have an important guiding role in agricultural production.

## Supporting information

S1 Raw images (DOCX)
