# Supplementary figures and images for: Potential risk of plant viruses entering disease cycle in surface water in protected vegetable growing areas of Eastern China
Source: PLoS One. 2023 Jan 25;18(1):e0280303. doi: 10.1371/journal.pone.0280303 (PMC9876373; doi:10.1371/journal.pone.0280303)

**Figure 1**


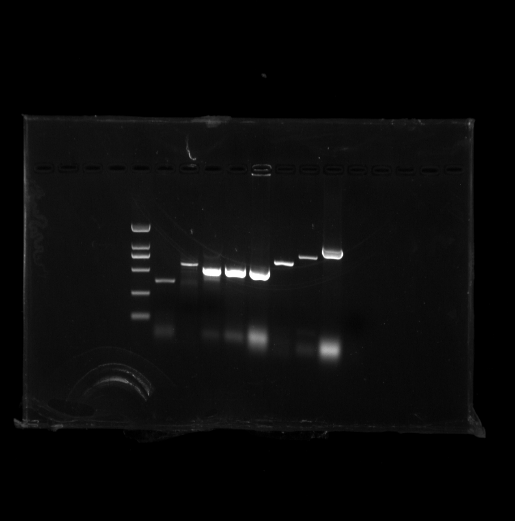


**M 1 2 3 4 5 6 7 8**

**Figure 2**


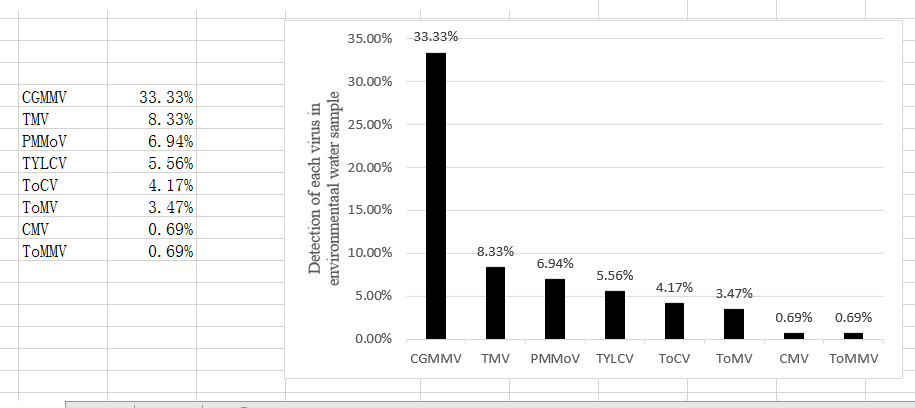


**Figure 3**


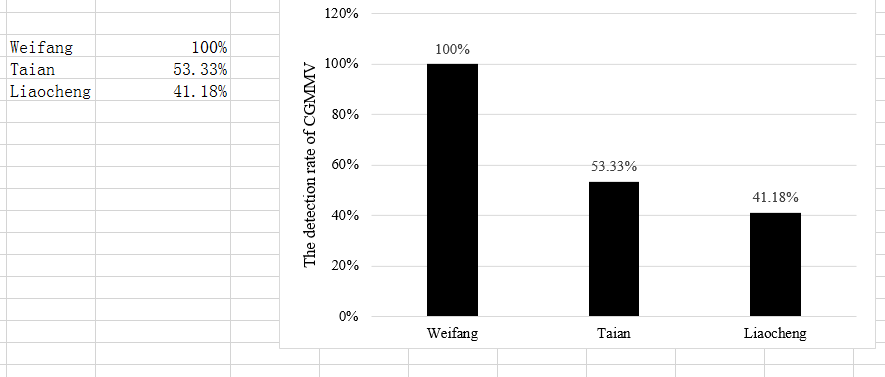


**Figure 4**


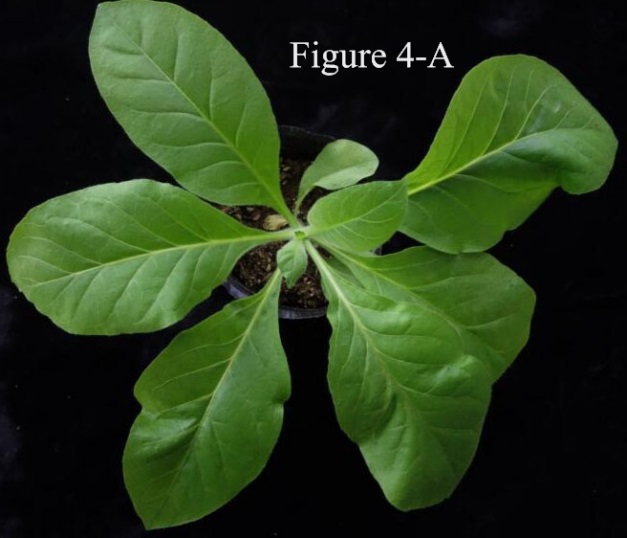


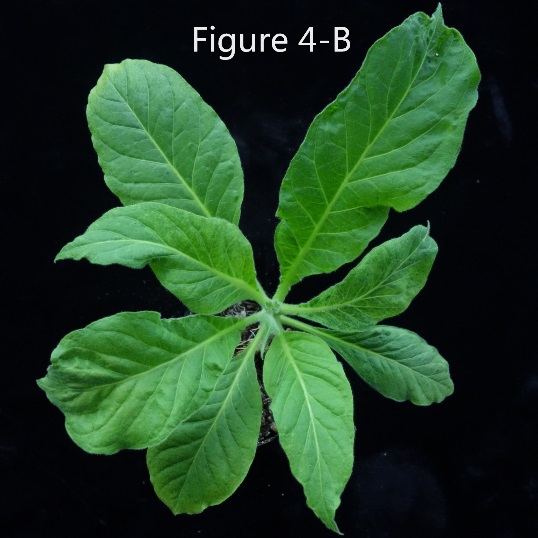


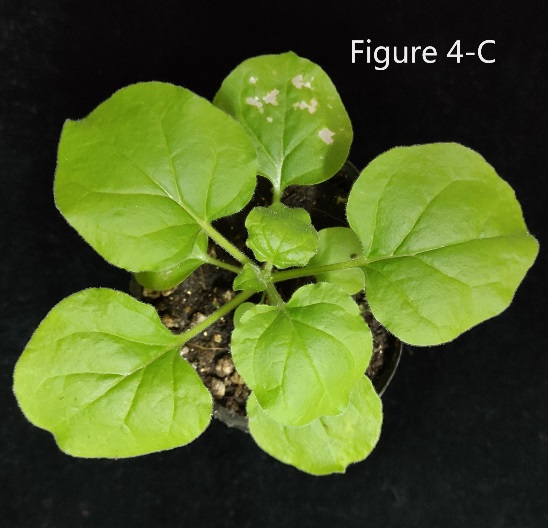

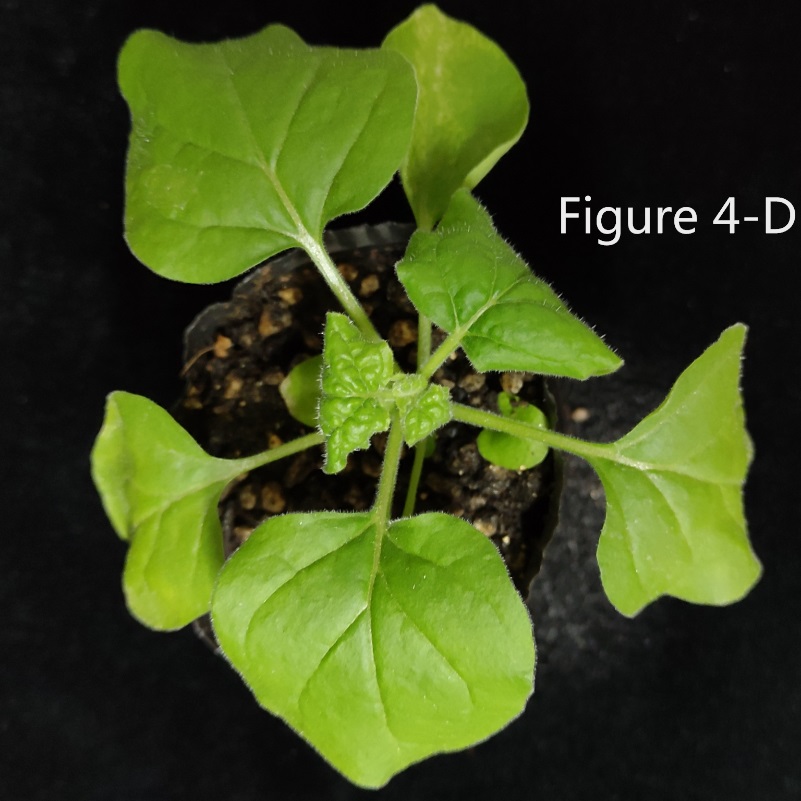


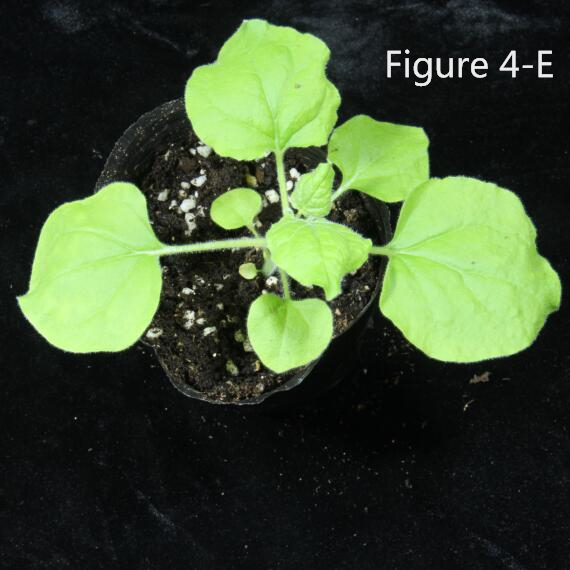


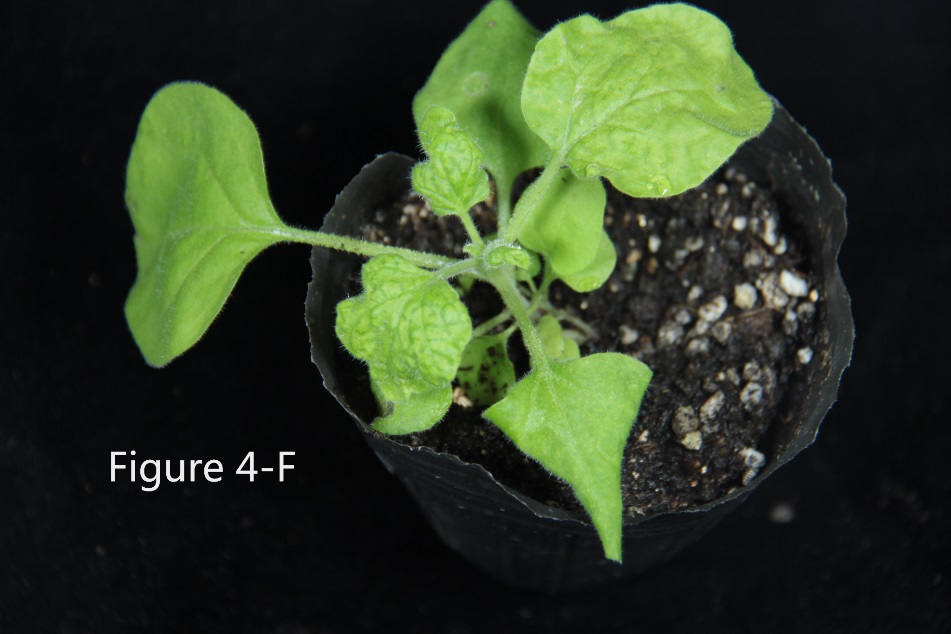


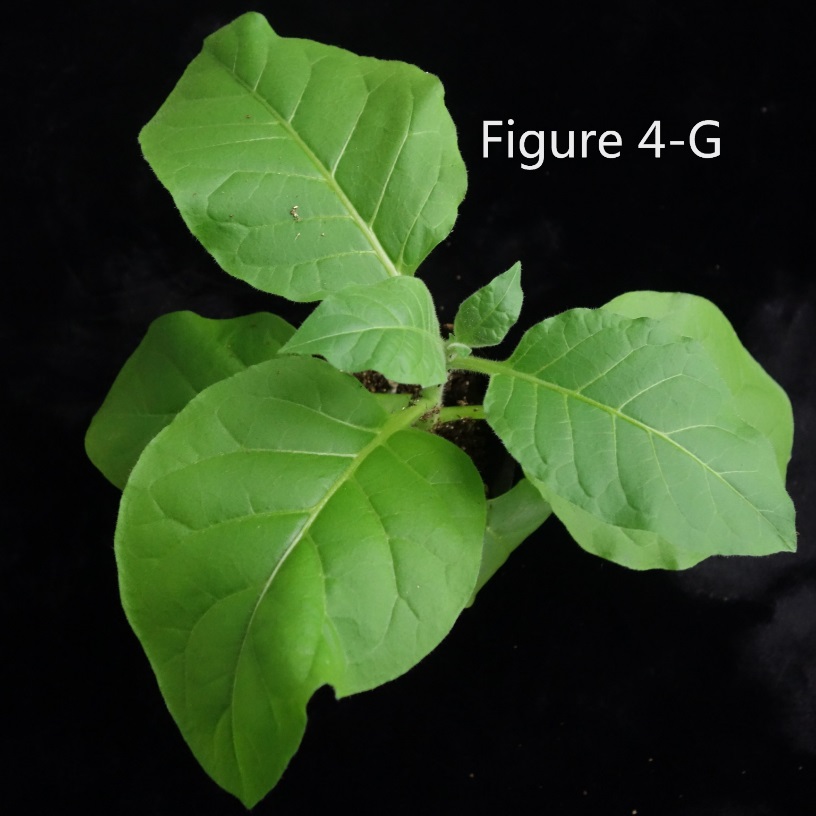


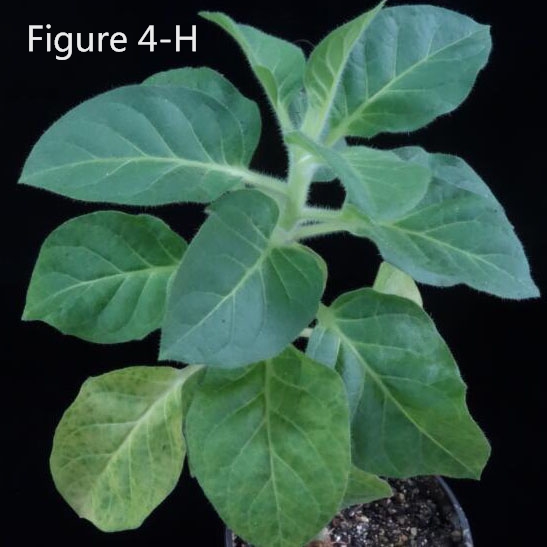


**Figure 5**


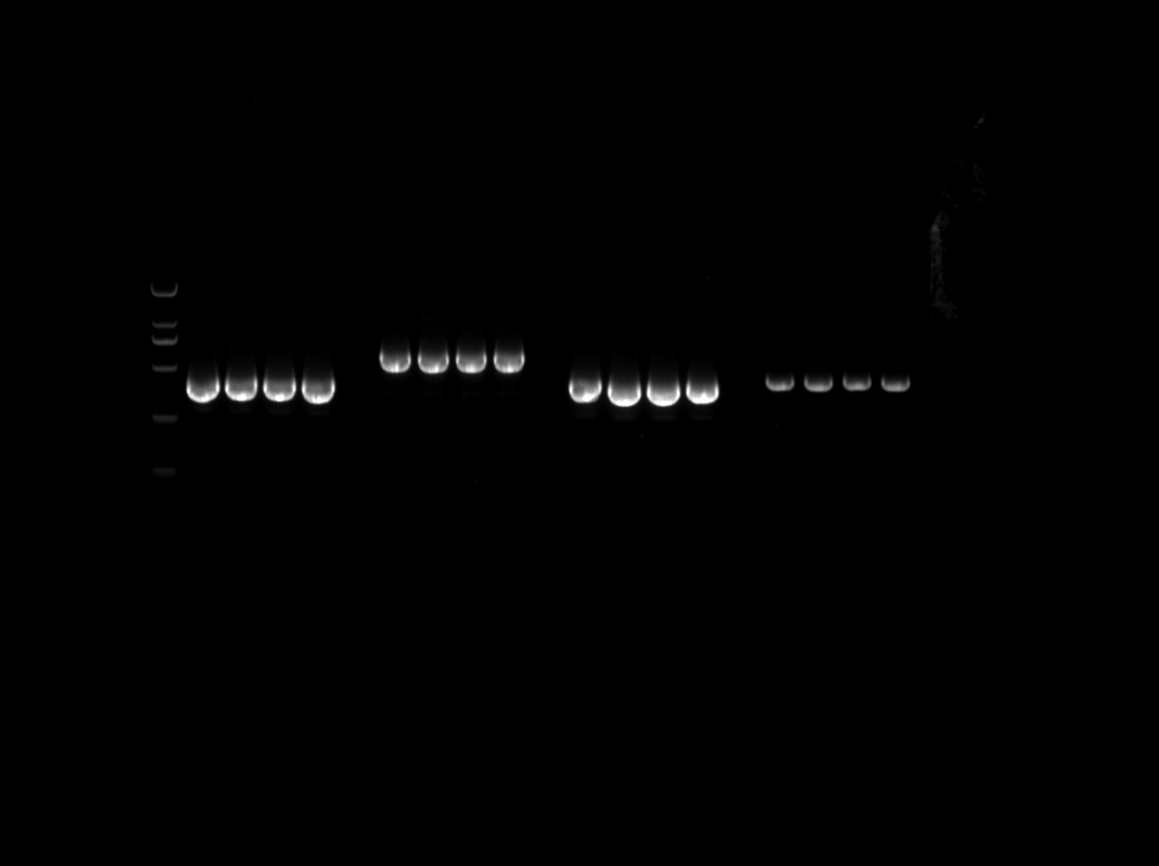


M 1 2 3 4CK 5 6 7 8 CK 9 10 11 12 CK13 14 15 16 CK

**Figure 6**


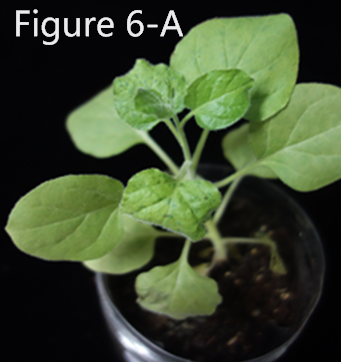


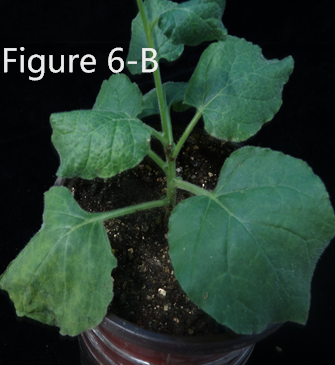


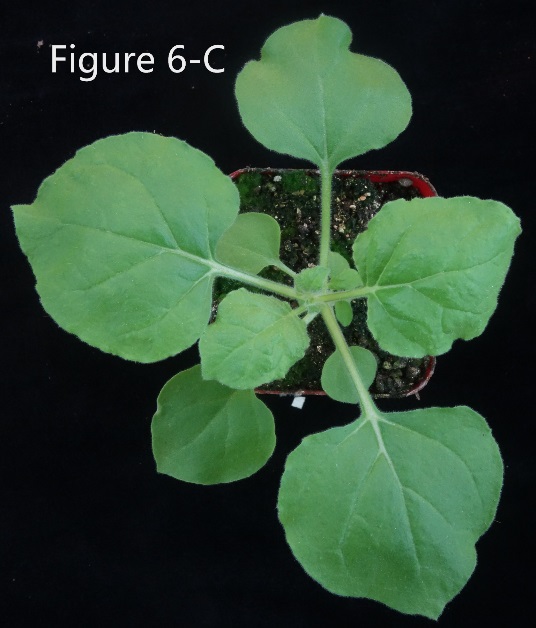


**Figure 7**


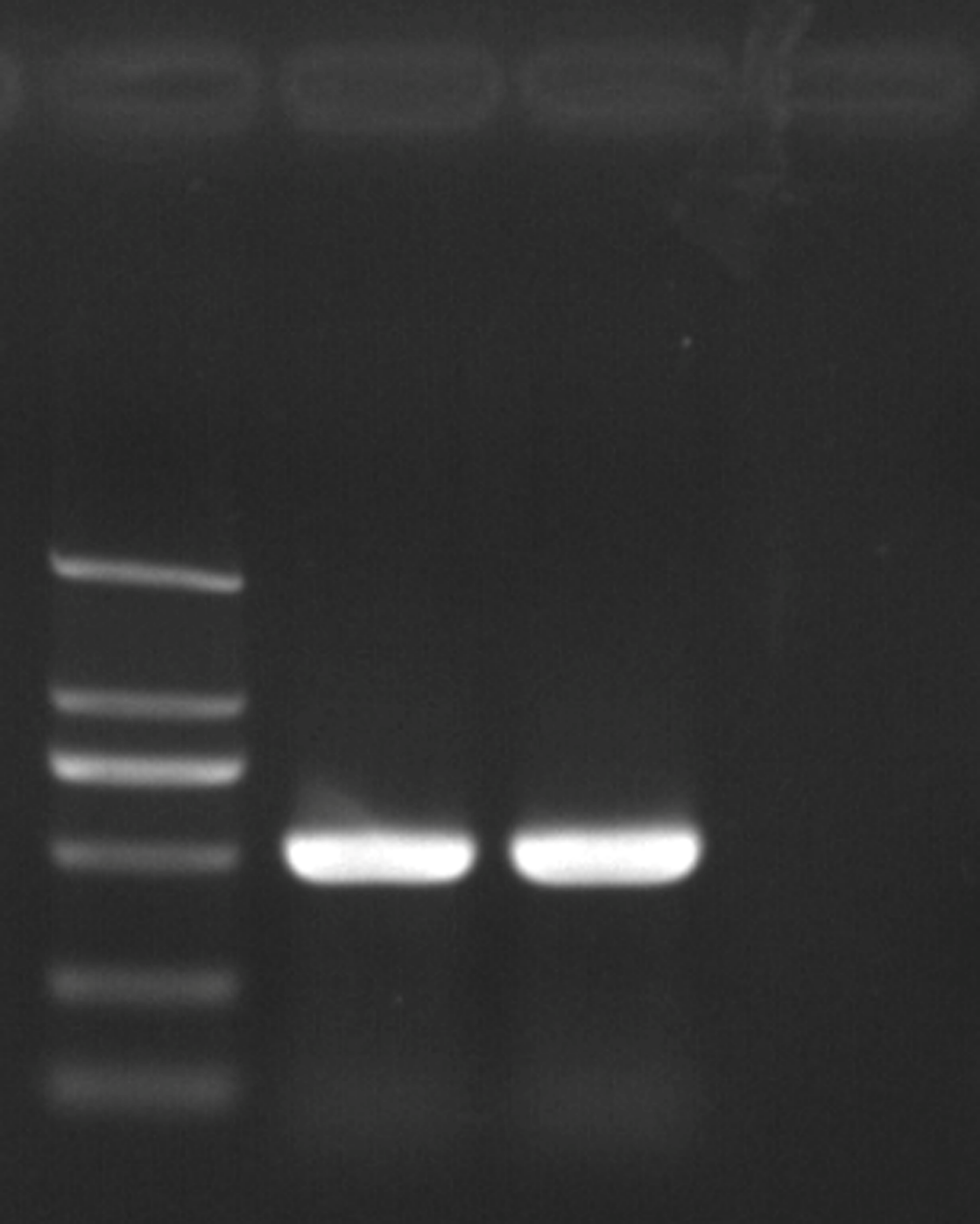


M 1 2 CK

Supplement: S1 Raw images — (DOCX) [file pone.0280303.s001.docx]
